# Frontiers and emerging trends of psychotherapy applications in anesthesiology: A comprehensive bibliometric analysis

**DOI:** 10.1016/j.jatmed.2024.12.002

**Published:** 2024-12-30

**Authors:** Yanyan Zhang, Huan Liu, Kaixin Wang, Yu Wang, Shiqian Huang, Pu Chen, Xinxin Yang, Yuanyuan Ding, Sanawaer Tuerhong, Li Huang, Xiangdong Chen

**Affiliations:** a Department of Anesthesiology, Union Hospital, Tongji Medical College, Huazhong University of Science and Technology, Wuhan 430022, China; b Institute of Anesthesia and Critical Care Medicine, Union Hospital, Tongji Medical College, Huazhong University of Science and Technology, Wuhan 430022, China; c Key Laboratory of Anesthesiology and Resuscitation (Huazhong University of Science and Technology), Ministry of Education, Wuhan 430022, China

**Keywords:** Psychotherapy, Anesthesiology, Bibliometrics, Research trends, Applications

## Abstract

**Background:**

Psychological therapy can mitigate preoperative negative emotions in patients and facilitate postoperative recovery, all while avoiding the side effects associated with sedative medications. However, there is currently a dearth of literature on the bibliometric analysis of the application of psychological therapy during the perioperative period of anesthesia. This study aims to assess the scientific achievements and emerging themes related to the application of psychological therapy in the domain of anesthesia.

**Methods:**

Retrieve studies on the application of psychological therapy in the field of anesthesia published from 2003 to October 31, 2023, from the Web of Science Core Collection database. Utilize VOS Viewer and CiteSpace to create visual bibliometric maps.

**Results:**

A total of 287 articles were included for analysis. Research on psychological therapy in the field of anesthesia has shown an increasing trend, particularly after 2011. The United States leads in terms of publication volume and collaborations. The *Journal of Perianesthesia Nursing* is the most prolific journal for publications, with Schreiber KL having the highest H-index. The most common keywords are *anesthesia* and *pain*, while aromatherapy has emerged as a newly focused therapy in recent years.

**Conclusions:**

Over the past two decades, psychological therapy has experienced rapid development. The United States has maintained a global leadership position in relevant research, making significant contributions. The focus of research has gradually shifted from the effectiveness of psychological therapy in controlling preoperative patient negativity to the development of various therapy methods and their impact on postoperative patient recovery.

## Introduction

With the advancement of medical technology and societal progress, there has been a shift in modern medical philosophy from a disease-centered approach to a patient-centered one.^1^ In the treatment process, attention is not only directed at the disease itself but also at addressing the psychological issues that arise during treatment.^2^ Many patients experience negative emotions such as anxiety and panic as surgery approaches.^3^, ^4^ The causes of such psychological stress reactions are diverse, with concerns and fears about anesthesia being particularly noteworthy.^5^ Studies have found that preoperative stress and anxiety in patients not only increase the sensitivity to postoperative pain perception and the use of analgesics but also, through psychoneuroimmunological mechanisms, can delay the healing of surgical incisions.^6^, ^7^, ^8^ Therefore, reducing preoperative negative emotions can bring about positive postoperative outcomes for patients.

Currently, the primary therapy for managing psychological stress reactions involves intravenous administration of analgesics or sedatives to attenuate the excitability of the sympathetic nervous system.^9^, ^10^ However, these drugs also come with side effects such as respiratory depression, intestinal obstruction, and delirium. This is especially concerning for pregnant women, where the choice of drugs is limited.^11^ The stress and anxiety experienced by the mother have profound implications for the psychological development of the fetus.^12^, ^13^ In the quest for new alternative therapies, psychological therapys such as music therapy and aromatherapy have emerged.

Psychological therapy plays an important role in improving the quality and effectiveness of anesthesia treatment in anesthesiology. As it is widely known, anesthesia treatment has a significant impact on the emotional and psychological state of patients, and any emotional or psychological instability within the patient can affect the outcome of anesthesia treatment. Psychological therapy can help patients regulate their emotions, alleviate anxiety and tension, and improve the effectiveness of anesthesia treatment. Specifically, it includes anxiety-relieving psychological therapy, helping the patient establish positive beliefs and attitudes, and promoting recovery.^14^, ^15^, ^16^

Bibliometrics, which integrates mathematics and statistics, allows for the quantitative analysis of the production and dissemination of research literature. It provides relevant information such as authors, journals, institutions, or countries, assisting researchers in delineating the research trajectory and hotspots in a specific field, and predicting research trends.^17^, ^18^

As a newly emerging alternative therapy, there is a lack of literature surveys regarding its current and future development trends. Therefore, this paper aims to apply the method of bibliometrics to quantitatively and visually analyze the application of psychological therapy in the field of anesthesia, revealing the current research status and hotspots, and further predicting new development trends. The study aims to address the research question regarding the current research status and hotspots, as well as emerging development trends, in the field of psychological therapy in anesthesia.

## Materials and methods

### Data source and search strategy

**Fig. 1:**
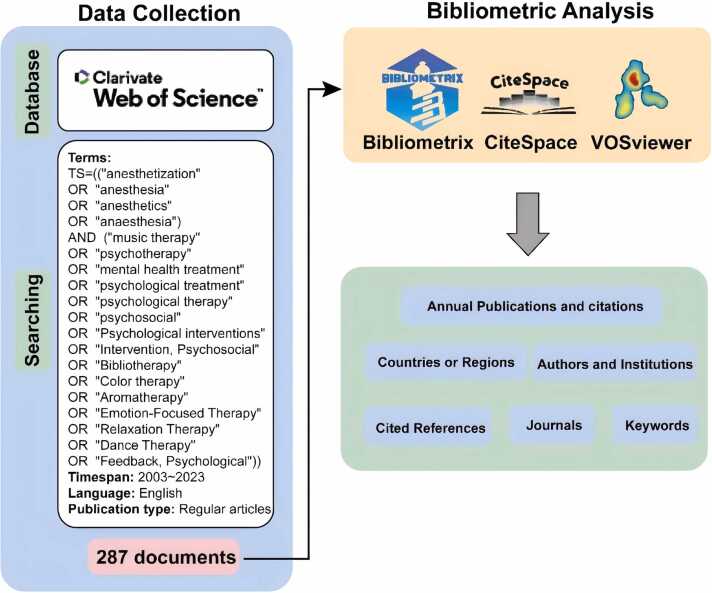
Flow-chart of the study.

To conduct the bibliometric analysis, the WosCC(Web of Science Core Collection) database was searched for articles published between 1 January 2003 and 31 October 2023 on the application of psychotherapeutic therapys in the field of anaesthesia. Compared with other databases such as Scopus, Medline and PubMed, Web of Science has a wider coverage of academic journals and is frequently used by researchers, providing the most comprehensive and reliable bibliometric analyses. The specific search strategy employed is outlined below: TS= ((“anesthetization” OR “anesthesia” OR “anesthetics” OR “anaesthesia”) AND (“music therapy” OR “psychotherapy” OR “mental health treatment” OR “psychological treatment” OR “psychological therapy” OR “psychosocial” OR “Psychological therapys” OR “therapy, Psychosocial” OR “Bibliotherapy” OR “Color therapy” OR “Aromatherapy” OR “Emotion-Focused Therapy” OR “Relaxation Therapy” OR “Dance Therapy” OR “Feedback, Psychological”)) AND “DOP = (2003–01–01/2023–10–31)”. For further analysis convenience, only articles written in English were selected. Subsequently, the search results were downloaded in plain text format, and basic information about the literature was extracted for subsequent research analysis. Fig. 1 illustrates the data collection and analysis process.

### Software for bibliometric analysis

This study employed scientometric methods to conduct statistical analyses on the collected literature data. Visualization tools such as VOSviewer 1.6.18 and CiteSpace (6.2.R4) were utilized for result visualization. The visual analysis in VOSviewer employs nodes, edges, colors, etc., to represent different meanings, providing an intuitive analysis of the research structure in a certain field from temporal and spatial perspectives.^19^ Nodes typically represent various research objects, such as authors, institutions, countries, etc., with their sizes often indicating publication quantity and academic impact. The edges and density between nodes reflect the closeness of collaborative relationships, while the thickness of edges directly represents the strength of connections.^19^ In this study, these tools were used to analyze collaboration among countries, institutions, and authors, as well as the co-occurrence of keywords. CiteSpace software exhibits strong advantages in delineating the evolution of disciplinary fields, highlighting research hotspots, and tracking frontier dynamics.^20^ In this research, CiteSpace was employed to identify countries that produced a substantial number of publications during specific periods, highly cited references, and keywords. Additionally, it was also used to generate relevant timelines.

## Results

### Annual growth trend of publications

**Fig. 2:**
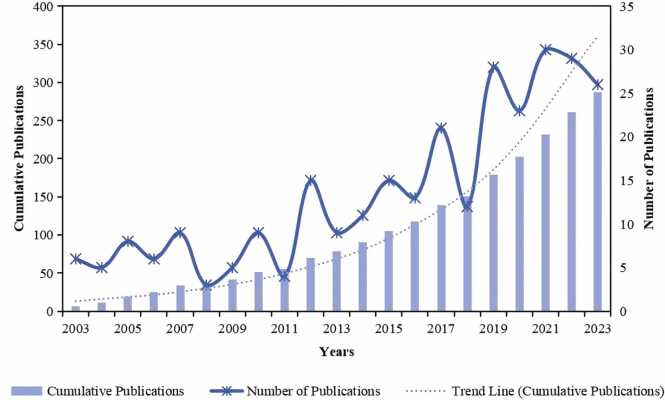
The number of articles about psychotherapy applications in anesthesiology per year from 2003 to 2023.

As depicted in Fig. 1, this study incorporated a total of 287 conventional articles on the application of psychological therapy in anesthesia. Fig. 2 illustrates the annual and cumulative publication counts. From the graph, it is evident that the number of publications has steadily increased, with a notable surge after 2011, reaching 15 publications for the first time in 2012. As of October 2023, a total of 26 relevant thematic articles have been published. The sharp increase after 2011 reflects the widespread application of psychological therapy in the field of anesthesia and the considerable attention it has gained in the academic community.

### Top institution

#### Contributions of countries or regions

**Fig. 3:**
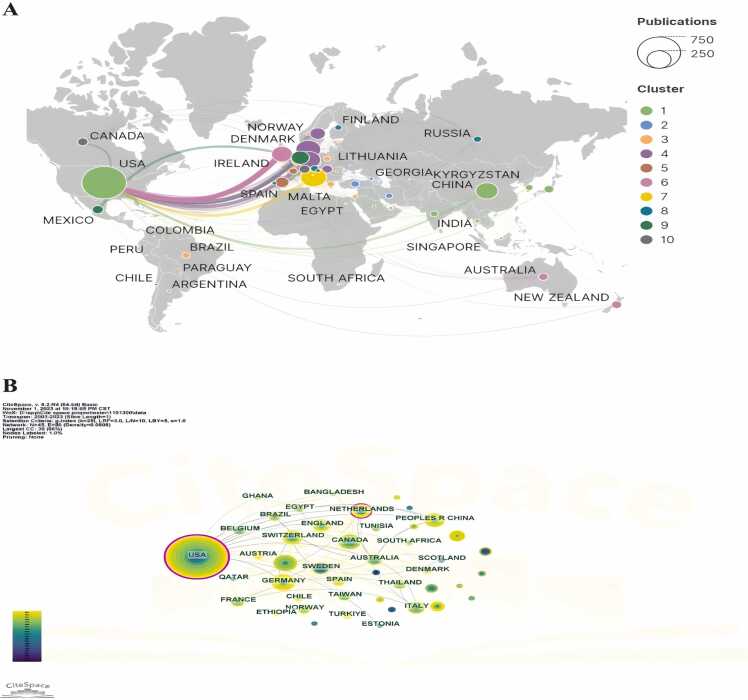
Country collaboration network in the field of psychotherapy applications in anesthesiology.

A total of 42 countries/regions participated in researching the role of psychological therapy in the perioperative period of anesthesia. Fig. 3 displays the national cooperation network, revealing that the United States (120 articles) leads in the number of publications, followed by China (23 articles), Canada (20 articles), Turkey (17 articles), and Germany (15 articles). The United States, with both the highest publication volume and centrality among all countries, signifies its leadership in this field. The collaboration between the United States and countries such as Ireland, Spain, and China is notable, while cooperation among other countries appears limited.

#### Contributions of institutions

**Fig. 4:**
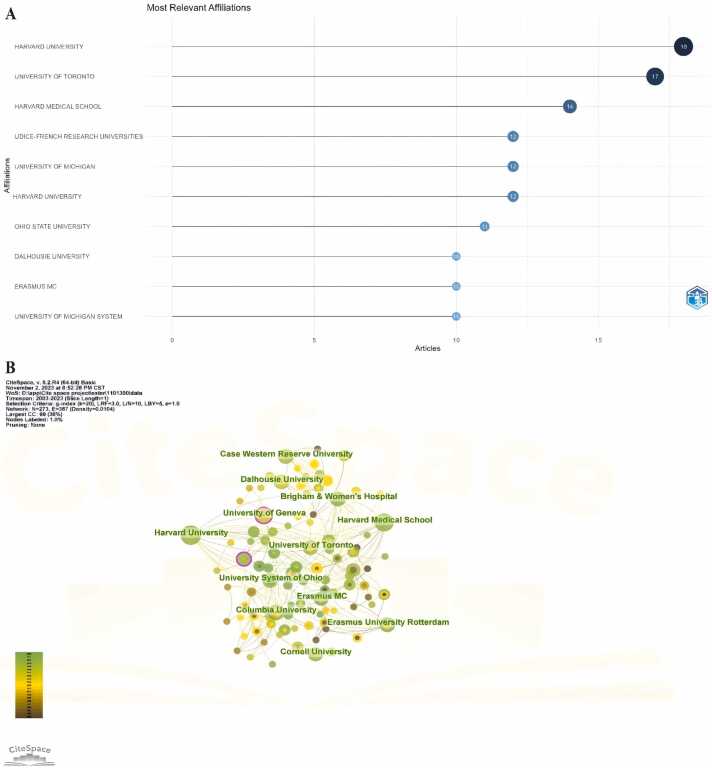
Contributions of Institutions. (A) Top ten institutions in the field of psychotherapy applications in anesthesiology. (B) Institution collaboration network.

A total of 593 different institutions participated in the publication of relevant research. Fig. 4A shows the top ten institutions in terms of publication volume. Harvard University is considered the most prolific institution, with a total of 18 published papers. Following closely are the University of Toronto (17 papers), Harvard Medical School (14 papers), and UDICE-French Research Universities, a consortium of ten prestigious French universities (12 papers). The University of Geneva and Ohio State University emerge as the most central institutions. As seen in Fig. 4B, schools within the same country tend to collaborate more with domestic institutions, possibly influenced by factors such as politics and the economy.

#### Analysis of journals

**Fig. 5:**
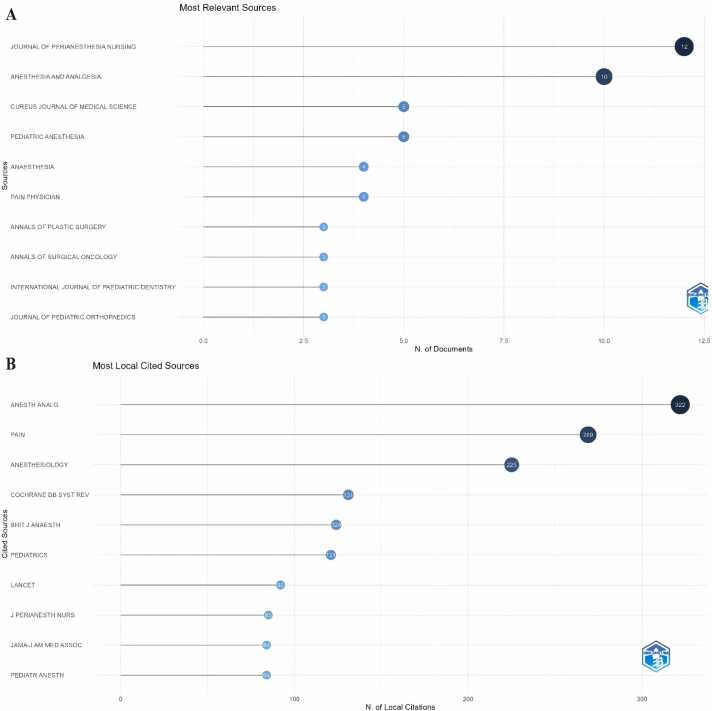
Analysis of Journals. (A) Top ten journals with the most published articles. (B) Top ten highly cited papers.

Fig. 5A lists the highest-yielding journals. The Journal of Perianesthesia Nursing (12 articles) stands out as the most prolific journal in this field, followed by Anesthesia and Analgesia (10 articles), Cureus Journal of Medical Science (5 articles), and Pediatric Anesthesia (5 articles). Among the top ten most productive journals, ANAESTHESIA has the highest Impact Factor (IF) at 10.7.

The impact of a journal is determined by the frequency of its citations, indicating whether the journal has had a significant influence on the scientific community. According to Fig. 5B, the most frequently cited journal is *Anesthesia and Analgesia* (322 citations), followed by *Pain* (269 citations), *Anesthesiology* (225 citations), and *Cochrane Database of Systematic Reviews* (131 citations).

#### Contribution of authors

**Fig. 6:**
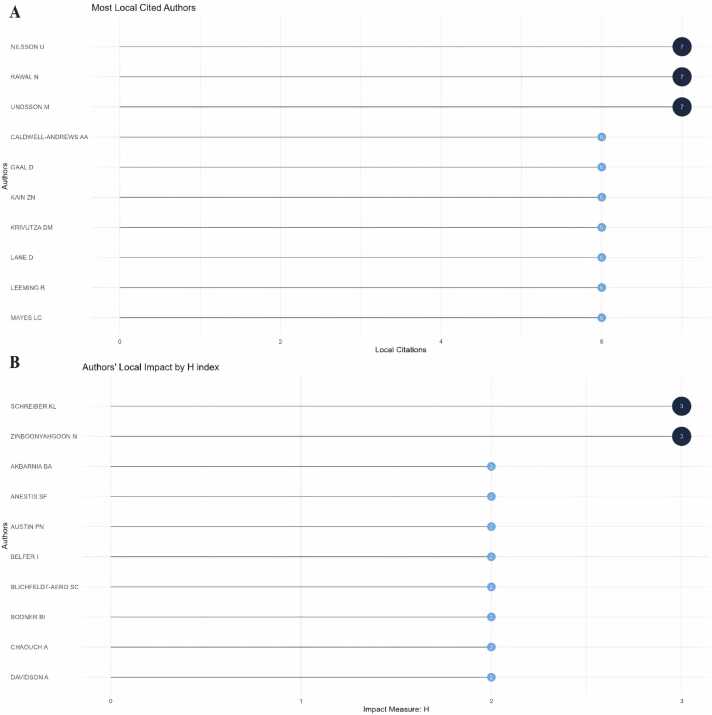
Contribution of Authors. (A) Top 10 authors with the highest citations. (B) Top 10 authors with the highest H index.

Fig. 6A presents the top ten authors ranked by citation count. Among them, Nilsson U, Rawal N, and Unosson M all hold the top position with seven citations each. Further analysis reveals that the top ten most cited authors are predominantly from European countries. Fig. 6B displays the ten authors with the highest H-index in the field, showcasing the influential figures such as Schreiber KL, Zinboonyahgoon N, Akbarnia BA, and Anestis SF. By delving into their publications, one can swiftly grasp the dynamics and frontiers of this field. Notably, Schreiber KL emerges as the most prolific author in terms of publication volume, while Akbarnia BA follows closely, signifying their leadership in the field.

### Keyword analysis

#### Frequency and clustering analysis of keywords

**Fig. 7:**
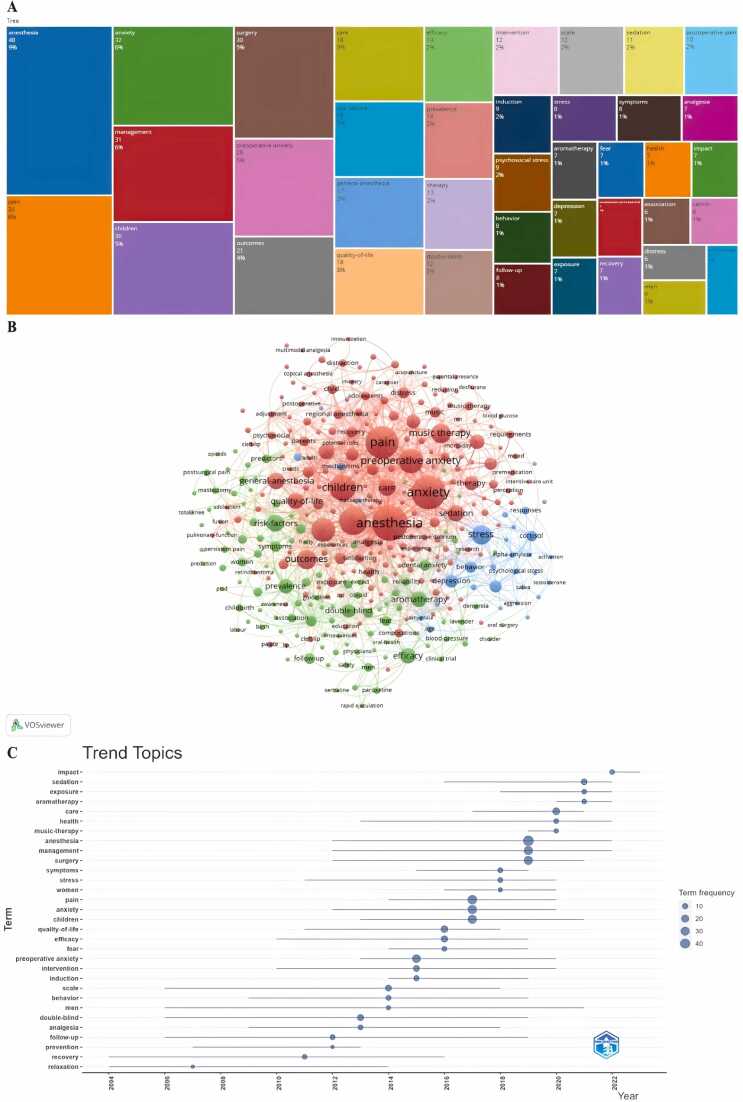
Analysis of keywords. (A) Hot keywords. (B) Keyword co-occurrence. (C) Key word time series diagram.

Keyword analysis provides a rapid insight into the status and developmental directions of a field. Based on the co-occurrence of keywords in VOSviewer, the most prominent keywords are anesthesia (48), followed by *pain* (34), *anxiety* (32), and *management* (31) (Fig. 7A). We constructed a network of keywords, yielding three distinct clusters (Fig. 7B). Cluster 1 (in red) includes *pain*, *anesthesia*, *anxiety*, *children*, *music therapy*, *quality-of-life*. Cluster 2 (in green) comprises *risk-factors*, *prevalence*, *double-blind*, *postsurgical pain*, *efficacy*, *fear*, *clinical trial*. Cluster 3 (in blue) consists of *stress*, *behavior*, *depression*, *activation*, *response*. A time-series graph was generated using VOSviewer to visually depict the evolution of research hotspots over time (Fig. 7C). It reveals that research focus has shifted from earlier topics, such as relaxation, recovery, and prevention, to more recent topics, such as anxiety, efficacy, and pain. Recently, the focus of research has further shifted to areas such as affect, aromatherapy, and sedation.

#### Analysis of keywords and citation bursts

**Fig. 8:**
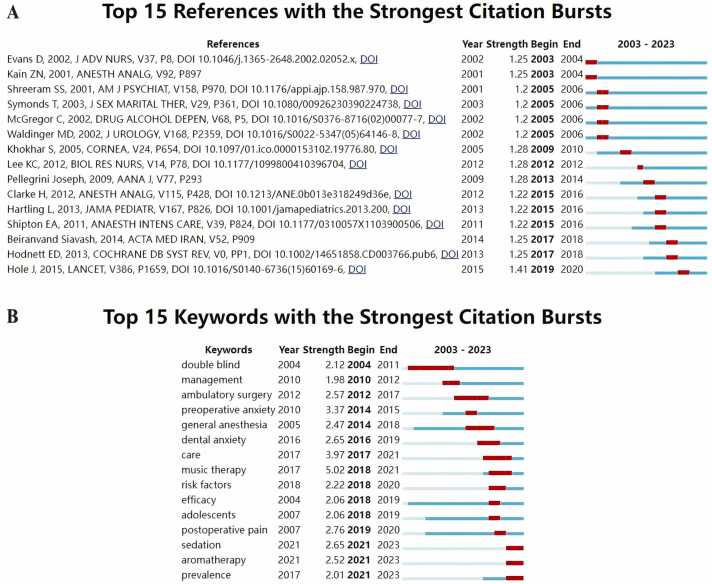
Analysis of citation bursts. (A) Top 15 references with the strongest citation bursts. (B) Top 15 keywords with the strongest citation bursts.

Using CiteSpace, we identified the 15 most reliable citation bursts in this field (Fig. 8A). The reference with the highest strength (1.41) is the work by Jenny Hole titled “*Music as an aid for postoperative recovery in adults: a systematic review and meta-analysis*,” mainly focusing on meta-analysis of music as an adjunct for adult postoperative recovery.^21^ Out of the 15 references, 10 were published between 2003 and 2023, indicating their sustained citation frequency over the past two decades. This suggests that research in this field will continue to receive attention.

In the keyword burst analysis, we particularly focused on the top 15 keywords experiencing bursts (Fig. 8B). These keywords represent the current research hotspots in the field and indicate potential directions for future studies, including sedation, aromatherapy, and prevalence.These bursts can provide valuable insights into the direction of future research in anesthesia psychology, including the potential for further investigation into the effectiveness of aromatherapy therapys in this context. Ultimately, continued exploration of these trends will help improve patient outcomes and advance the field of anesthesia psychology.

## Discussion

As depicted in Fig. 1, a total of 287 relevant articles were included in the analysis. Fig. 2 illustrates the annual and cumulative counts of publications related to psychological therapy in the field of anesthesia. Over the past two decades, the research development of psychological therapy in anesthesia can be delineated into two phases: “Fluctuation (2003–2011)” and “Steady Growth (2012–2023)”. During 2003–2011, there was a relatively modest output of publications. Scholars gradually began to take notice of psychological therapys in anesthesia during this period, with pioneering researchers entering the field. With the accumulation of related studies, psychological therapy gained growing concern. In 2012, there was a surge in publications, surpassing 10 for the first time. This marks the onset of rapid development and entry into the “Steady Growth” phase. This explosive growth is likely correlated with the maturation of the concept of patient comfort in medical care. The 2012 pain management guidelines suggest that opioids should be used as a last resort for pain management. Non-pharmacological treatments are also described in the article. It is evident that there is a growing awareness of the need to focus not only on the resolution of pain itself, but also on the side effects of therapeutic measures.^22^ With advancements in medical practices and anesthesia technology, patients have shifted from the basic need for pain relief to a higher pursuit of comfort in healthcare, emphasizing mental satisfaction and humane care.^23^ It is conjectured that research related to psychological therapy will continue to grow in the coming years.

The top 10 countries collectively contributed 211 articles, constituting 73.5 % of all publications. Among these nations, the United States holds a dominant position in terms of publication quantity, standing at the forefront globally. This is likely linked to the country’s developed economic conditions and healthcare environment. As early as 1992, American nursing expert Catherine proposed the theory of comfort-oriented medical care, emphasizing the dual comfort of patients both physiologically and psychologically during treatment. In 2004, Katherine again summarised the application of the comfort theory in the perianesthesia environment.^24^ Notably, China and Germany have been particularly active in this field in recent years, with China ranking second with 22 publications. This may be attributed to the growing interest of Chinese researchers in this field, along with an increase in academic research funding.^25^

The most productive top 10 institutions are located in developed countries, primarily in the United States, Canada, and the Netherlands. This highlights the crucial role of economic foundations in supporting scientific research. Despite China ranking second in the quantity of publications, no Chinese institution has entered the top ten. In 1999, Abramowitz explored the relationship between economic growth and technological innovation in different countries. As the findings showed, the developed economies were able to achieve success in the field of science and technology because their economic foundations could provide funding and other resources to support scientific research and technological innovation.^26^ Additionally, in the analysis of collaborative institutions, Case Western Reserve University exhibits the strongest collaborative intent, maintaining partnerships with multiple institutions.

As keywords reflect the core content of a study, co-occurrence analysis can identify high-frequency keywords appearing in different studies, aiding researchers in swiftly grasping research hotspots.^27^ In this study, the most common keyword is *anesthesia*, with a frequency of 48. Following closely are *pain*, *anxiety*, and *management*. Additionally, *children* is a focal research term. Due to their immature physiology and psychology, children exhibit varying degrees of perioperative anxiety, particularly in unfamiliar environments or when separated from parents.^28^ This psychological stress not only affects postoperative recovery but also hampers long-term cognitive and emotional development in children.^29^, ^30^, ^31^ Therefore, children are a primary target for perioperative psychological therapys. Numerous studies suggest that diverting a child’s attention effectively reduces anxiety levels. This is achieved through methods such as clown figures, storytelling, watching videos or playing games, and toy transport vehicles to comfort the child.^32^, ^33^, ^34^ However, the current bias and low quality in relevant research make the evidence less robust. The effectiveness of these methods in significantly reducing children’s psychological stress awaits confirmation from high-quality clinical evidence. Parental presence during induction is also an available option. For many children, the presence of parents is crucial, and being in an unfamiliar environment without parental companionship undoubtedly intensifies inner anxiety. Literature from the 1960s reported maternal accompaniment during anesthesia induction for children.^35^ Still, the effectiveness of this method in alleviating anxiety remains controversial.^36^ A systematic review by Manyande and others suggests that accompanied induction does not necessarily lower preoperative anxiety levels in children.^37^

Through the analysis of the keyword timeline, developmental trends can be anticipated. Early research primarily focused on exploring the effectiveness of psychological therapy in soothing patient emotions under different anesthesia methods, whereas current studies are more centered on examining postoperative outcomes following psychological therapy treatments, particularly the impact on postoperative pain. Keyword burst analysis also aids in identifying potential research hotspots. Aromatherapy, as a recently emerged therapy, represents a new direction for future research.^38^ Fragrances can transmit neural impulses to the olfactory bulb, and consequently trigger the release of various neurotransmitters in the brain, thereby alleviating stress, relieving pain, and ultimately leading to physiological and psychological changes.

Surgical operations bring patients a lot of psychological and physiological stress stimuli. The focus of research has gradually shifted from the effectiveness of psychological therapy in controlling preoperative patient negativity to the development of various therapy methods and their impact on postoperative patient recovery. Non-pharmacologic therapy as an adjunctive treatment can relieve the emotional stress and anxiety of the perioperative period, reduce the amount of painkillers used, and improve postoperative rehabilitation quality. Currently, with the development of the Enhanced Recovery After Surgery (ERAS) concept, multimodal interventions to improve patient recovery have become a trend. Non-pharmacologic therapies such as music therapy and aromatherapy, due to their many advantages such as affordability, non-invasiveness, and ease of implementation, will become important adjunct measures to improve the quality of the perioperative period.^39^, ^40^, ^41^, ^42^

This study has certain limitations. Firstly, we only analyzed English articles from the Woscc database, which might result in the omission of some publications. Secondly, due to the continuous updating of databases, the results of bibliometric analysis usually lag behind the actual research landscape. There may be potential biases, especially in the methodology section, such as a reliance on citation counts (which may favor older publications) or regional biases in the databases used. Despite these limitations, this study significantly aids researchers in quickly understanding the current status and future trends of psychological therapy in the field of anesthesia, proposing further research directions.

## Conclusions

Our study pioneers the use of bibliometric analysis to explore the current status and emerging global trends in the application of psychological therapy treatments in the field of anesthesia. The focus of research has gradually shifted from the effectiveness of psychological therapy in controlling preoperative patient negativity to the development of various therapy methods and their impact on postoperative patient recovery. Overall, the utilization of psychological therapy in anesthesia is in a phase of rapid development, and this research domain is likely to continue expanding swiftly. In the future, as the concept of patient-centered care gains greater prominence, the management of perioperative anxiety is poised to receive increased attention. The value of non-pharmacological therapeutic therapys in this field will also be more thoroughly explored and appreciated. The study unveils a shift in focus within this field—from the effectiveness of psychological therapy in controlling preoperative negative emotions to the development of various therapy methods and their impact on postoperative patient recovery. Anesthesia psychological therapy can offer clinical doctors and researchers a range of practical recommendations, including strategies for preoperative emotional regulation, psychotherapy techniques, and postoperative pain management and recovery. These suggestions assist them in effectively addressing patients’ psychological and emotional health concerns, enhancing their surgical experience and treatment outcomes. Additionally, a significant disparity in this field is identified, notably manifested in the substantial gap between developed and developing countries. Therefore, researchers newly entering this domain can gain a quick and comprehensive understanding of its evolution and frontiers through this paper.

## CRediT authorship contribution statement

**Kaixin Wang:** Writing – original draft, Data curation, Conceptualization. **Yu Wang:** Software, Resources, Data curation, Conceptualization. **Shiqian Huang:** Methodology, Investigation, Formal analysis, Conceptualization. **Pu Chen:** Investigation, Formal analysis, Conceptualization. **Xinxin Yang:** Software, Resources. **Yuanyuan Ding:** Software, Resources, Project administration, Methodology, Conceptualization. **Li Huang:** Methodology, Investigation, Formal analysis. **Xiangdong Chen:** Writing – review & editing, Supervision, Project administration, Conceptualization. **Yanyan Zhang:** Writing – review & editing, Writing – original draft, Software, Resources, Project administration, Methodology, Investigation, Formal analysis, Data curation, Conceptualization. **Huan Liu:** Writing – review & editing, Writing – original draft, Methodology, Investigation, Formal analysis, Data curation, Conceptualization.

## Disclosure statement

Not applicable.

## Ethical statement

Not applicable.

## Funding

This study was supported by the National Key Research and Development Program of China (grant 2018YFC2001802 to XC); the 10.13039/501100001809National Natural Science Foundation of China (grant 82071251 to XC); Hubei Province Key Research and Development Program (grant 2021BCA145 to XC).

## Declaration of competing interest

The authors declare that they have no known competing financial interests or personal relationships that could have appeared to influence the work reported in this paper.

Xiangdong Chen is an Editor-in-Chief for *Journal of Anesthesia and Translational Medicine* and was not involved in the editorial review or the decision to publish this article.

## Data Availability

All study data are included in the article.
